# Deep venous thrombosis after Achilles tendon rupture is associated with poor patient-reported outcome

**DOI:** 10.1007/s00167-020-05945-2

**Published:** 2020-04-20

**Authors:** Simon Svedman, Gunnar Edman, Paul W. Ackermann

**Affiliations:** 1 Integrative Orthopedic Laboratory, Department of Molecular Medicine and Surgery, Karolinska Institutet, Karolinska Universitetssjukhuset, 171 76 Stockholm, Sweden; 2 Department of Orthopedics, Karolinska University Hospital, Stockholm, Sweden; 3 Department of Psychiatry, Tiohundra AB, Norrtälje, Sweden

**Keywords:** Achilles tendon, Deep venous thrombosis, Immobilization, Patient reported outcome measures, Venous thromboembolism

## Abstract

**Purpose:**

The aim of this study was to investigate whether patient subjective and functional outcomes after Achilles tendon rupture (ATR) are related to deep venous thrombosis (DVT) during leg immobilization.

**Methods:**

A cohort study with prospectively collected randomized data was conducted between 2010 and 2017. Two-hundred and fifty-one Patients with an Achilles tendon rupture (mean age = 41 ± 8), treated with uniform surgical techniques, were retrospectively analyzed. DVT incidence at 2 and 6 weeks was assessed using compression duplex ultrasound. At 12 months patient-reported outcomes were assessed using the Achilles tendon Total Rupture Score (ATRS), Foot- and Ankle Outcome Score (FAOS), Physical Activity Scale (PAS) and functional outcome with the calf-muscle endurance test. ANOVA analyses were used and adjusted for assumed confounding factors (patient age, sex, BMI and rehabilitation).

**Results:**

The total DVT incidence was 122 out of 251 (49%). Patients suffering a DVT exhibited significantly lower ATRS at 1 year compared to patients without DVT (mean 76 vs 83, 95% CI 71–79 vs 80–87; *p* < 0.01). Sixty-seven percent (95% CI 57–77%) of the patients devoid of DVT reported a good outcome (ATRS > 80) compared to 51% (95% CI 41–61%) of the patients sustaining a DVT (*p* < 0.05). Quality of life displayed significantly better outcome in the non-DVT versus DVT patients (mean = 75 (95% CI 71–79) vs. mean = 68 (95% CI 64–72); *p* < 0.05). A significant difference in total concentric work was observed between non-DVT and DVT patients (median = 1.9 kJ (IQR = 0.9 kJ) vs. median = 1.6 kJ (IQR = 1.0 kJ); *p* < 0.01).

**Conclusion:**

Sustaining a DVT during leg immobilization significantly impairs patient-reported outcome at 1 year after surgical repair of ATR.

**Level of evidence:**

III.

## Introduction

Immobilization of the lower limb is a considerable risk factor for venous thromboembolism, i.e., deep s(DVT) and pulmonary embolism (PE). The incidence of DVT during leg immobilization ranges between 4.3% and 40% in a wide field of orthopedic conditions, and even up to 50% after, e.g., Achilles tendon rupture (ATR), irrespective of surgical or non-surgical treatment [9, 39].

The efficacy and optimal thromboprophylaxis regimen during leg immobilization, especially in high-risk patients such as ATR, are largely unclear [1, 9, 11, 22, 39]. Current guidelines do not recommend general DVT prophylaxis during lower-limb immobilization, but rather suggest individual risk assessment [1, 11, 12]. Aside from the risk of DVTs to propagate and cause PE, impaired subjective outcome with symptoms such as limb swelling and pain may cause considerable long-term morbidity.

Long-term patient-reported outcome in patients with an Achilles tendon rupture is largely variable and underlying causes have not been fully elucidated. Moreover, the clinical significance of asymptomatic DVT is unknown and the Cochrane Library requests studies that investigate the long-term outcome of asymptomatic DVTs in patients with lower leg immobilization [39]. A recent study demonstrated that asymptomatic distal DVTs exhibit up to 72% risk of PE, which indicates a higher systemic impact than earlier believed [19]. However, whether suffering a distal DVT during leg immobilization can influence patient long-term outcome is unknown and such information could potentially change indications for anticoagulant prophylaxis.

In the present study, it was hypothesized that the occurrence of a DVT during leg immobilization would have negative effects on subjective outcome and on the functional calf-muscle endurance test. Since patients with an Achilles tendon rupture exhibit a high incidence of DVT and their patient-reported outcome varies, this is a good patient population to study the morbidity of leg immobilization.

## Material and methods

## Ethical approval

Ethical approval was obtained from the Regional Ethical Review Committee in Stockholm, Sweden (Dnr: 2009/2079–31/2 and Dnr: 2013/1791–31/3). All participants received oral and written information about the study procedure and provided written informed consent prior to surgery. Patients were given both written and oral information before signing the informed consent form.

### Patients

**Fig. 1:**
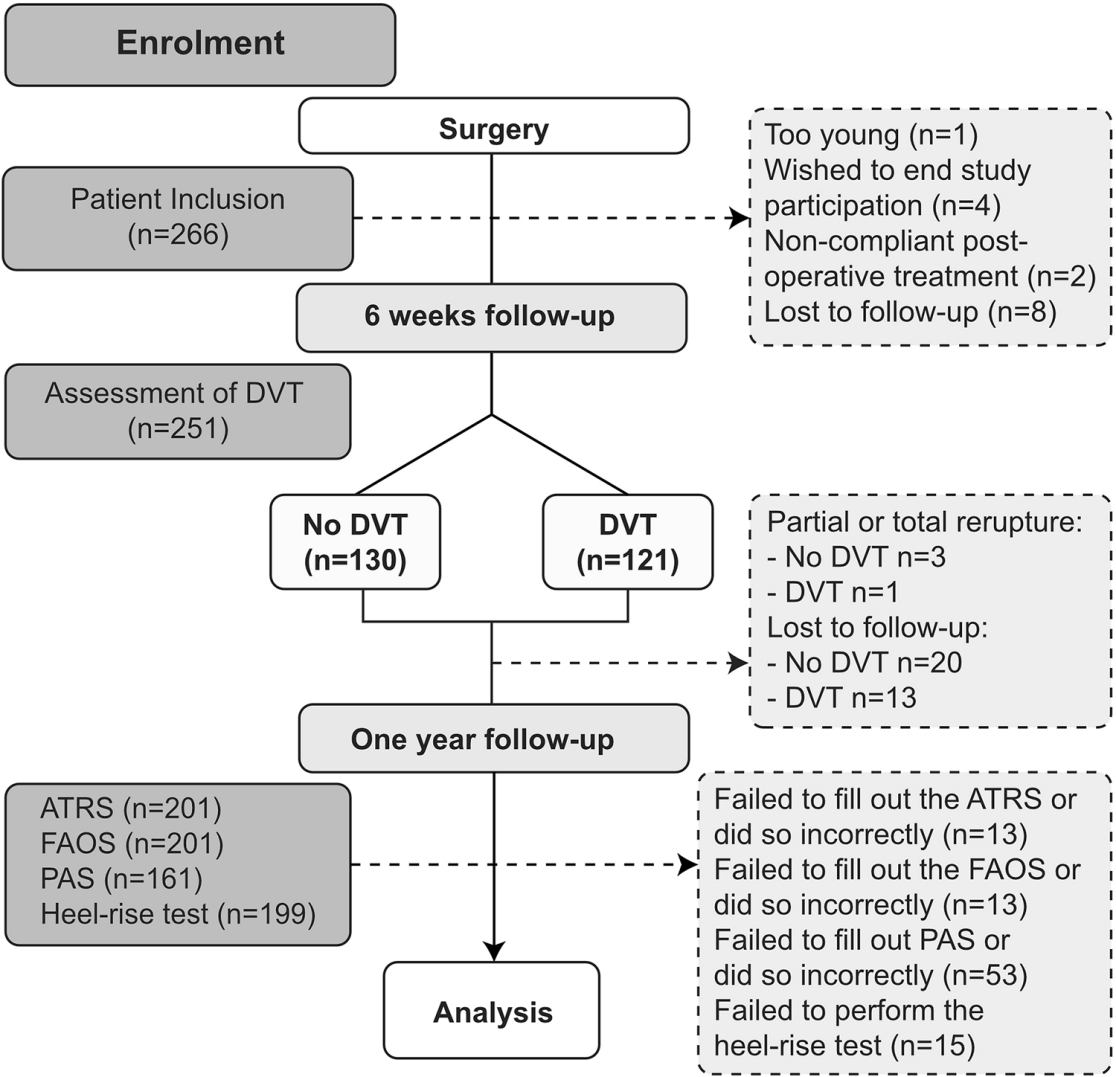
Patient flowchart. Two-hundred and sixty-six patients sustaining and operated on for unilateral Achilles tendon rupture were eligible for inclusion. After DVT screening, 251 patients were divided into the DVT and non-DVT group. Patient functional performance and patient questionnaires were assessed at the 1-year follow-up. *DVT *deep venous thrombosis

Data from 266 patients who had undergone surgical repair after acute Achilles tendon rupture were assessed and patients who had undergone compression duplex ultrasound at 2 or 6 weeks were retrospectively analyzed (Fig. 1). All patients had participated in three different randomized trials conducted between 2010 and 2017 at Karolinska University Hospital, Stockholm [3, 9, 10]. Detailed information about the trials is found below. Inclusion criteria in all studies were: patients with an acute Achilles tendon rupture with an age between 18 and 75 years. Exclusion criteria in all studies were: previous rupture of the same Achilles tendon, current anticoagulation treatment (including high-dose acetylsalicylic acid), known kidney failure, heart failure with pitting edema, thrombophlebitis, thromboembolic event during the previous 3 months, known malignancy, known hemophilia or thrombophilia, pregnancy, other surgery during the previous month, inability to follow instructions or planned follow-up at another hospital. Two-hundred and fifty-one patients with valid compression duplex ultrasound were assessed in the study (Fig. 1).

### Baseline patient characteristics

Baseline patient characteristics are presented in Table 1.

**Table 1:** Patient demographics. Demographics for 251 patients with acute Achilles tendon rupture and with or without deep venous thrombosis

Characteristic		No DVT (*n* = 129)	DVT (*n* = 122)	*p*-value
Sex				
Male	*n* (%)	103 (80)	103 (84)	n.s
Female	*n* (%)	26 (20)	19 (16)	n.s
Age, years	M (SD)	38 (7)	43 (8)	< 0.001
Body mass index	M (SD)	26 (3)	27 (4)	n.s
Smoker, yes	*n* (%)	6 (5)	7 (6)	n.s
Operative time, hh:mm	M (SD)	0:40 (0:14)	0:39 (0:15)	n.s

*DVT *deep venous thrombosis

### Surgical procedure and rehabilitation

All patients were operated on according to a standardized open surgical procedure, using a modified Kessler suture, as described earlier [10, 34], at Karolinska University Hospital. Patients were operated for their injury as soon as possible. Postoperatively, patients were prospectively randomized to four different leg immobilization protocols during the first 2 weeks. One trial investigated the effect of foot intermittent pneumatic compression (IPC) under a plaster cast on DVT frequency compared to plaster cast without IPC [10]. The second trial investigated differences in DVT incidence between patients with a calf IPC for 2 weeks underneath an orthosis compared to plaster cast [9]. The third trial investigated early mobilization in a weight-bearing orthosis compared to plaster cast treatment [36]. No patient received anti-thrombotic chemoprophylaxis before or after surgery, or during the rehabilitation period, as this would interfere with the incidence of thrombotic events, which was the primary outcome variable in all of the RCTs.

### DVT screening

Patients in all trials were assessed at 2 and 6 weeks postoperatively for DVT using unilateral compression duplex ultrasound (CDU), performed by two skilled sonographers using a Philips CX 50 ultrasound machine (Philips Medical Systems, Andover, MA, USA). The standard procedure included evaluation of all deep proximal and distal veins, including muscle veins, as well as vena saphena magna. If a thrombus was identified, the patient was noted for a DVT and the thrombus was categorized as proximal if located in or above the popliteal vein, distal if located in the deep veins of the tibialis posterior or fibularis vein and as a muscle vein thrombosis if located in perforator veins of the lower leg. The criteria for DVT diagnosis and the diagnostic procedure have been described earlier [9, 20]. Briefly, the DVT diagnosis was based on a transversal ultrasound compression test of the blood vessel, and assessment of blood flow in the veins by color Doppler flow. The CDU scans were re-evaluated after completion of the study by another ultrasonographer blinded to the treatment allocation. The accuracy of ultrasound in diagnosing DVT for patients without symptoms has for whole leg DVT by meta-analyses been shown to have a pooled sensitivity of 61% (95% CI 48–74%) and a pooled specificity of 95% (95% CI   91–98%), with an accuracy of 91.5% for ultrasound overall [40] [35].

The symptomatic or asymptomatic nature of the DVTs were not recorded in the trials, since it has been demonstrated to be difficult for the clinician to differentiate between the pain of the rupture or operation and pain that could be caused by a DVT [25]. If a DVT was diagnosed, patients were initially treated with low molecular weight heparin (200 units/kg) subcutaneously once daily and referred to an outpatient anticoagulation clinic for further investigation and treatment. Patients subjected to a DVT were instructed to start physical therapy in the same way as those without a DVT.

### Outcome measurements

At 1 year follow-up, the subjective outcome was measured using validated Likert scale questionnaires. The Achilles tendon Total Rupture Score (ATRS) Sweden, version 6, is validated and widely used in ATR research [24], where patients are asked ten questions about their leg limitations after injury (10 = no limitations). A difference of 7–10 points in ATRS between patient groups has been considered clinically significant [13, 24]. Patients with ATRS > 80 were categorized as having a good outcome [33]. The Foot and Ankle Outcome Score (FAOS) [30] was additionally used to let patients describe symptoms not available in the ATRS. To grade the patients’ physical activity level, the Physical Activity Score (PAS) was used [26]. The PAS was initially used at the 2-week follow-up about physical activity level before injury, and then again at 1 year.

Patients’ functional outcomes were evaluated at 1 year, using an established validated regimen of the heel-rise endurance test using Muscle Lab (Ergotest Technology, Oslo, Norway) [23, 32]. The heel-rise test was supervised by a trained physical therapist.

### Statistical analysis

All data were analyzed using SPSS (IBM SPSS, version 24.0. Armonk, NY, USA). The sample size was calculated on a minimal clinical difference in ATRS of 7 points between the patients with and without DVT, and a standard deviation of 15 points. It was determined that a sample size of 73 patients per group would be necessary to detect a seven-point ATRS difference with 80% power when alpha was set equal to 5%. One-hundred and forty-six patients in total were needed and we included 251 patients to account for loss at 1-year follow-up and for subgroup analyses.

All variables were checked for skewness and outliers. Patient characteristics were summarized with frequency, mean and standard deviation when appropriate, and compared for differences between the groups using Student’s *t* test or Chi-square test. Group comparison for parametric outcome variables were analyzed using an ANOVA method to control for confounding factors such as patient characteristics (age, BMI, gender), smoking, duration of operative time and postoperative treatment, and are presented with mean value and 95% confidence interval. Group comparison for non-parametric outcome variables were compared using the Mann–Whitney *U* test and are presented with median and interquartile range. Adjusted and non-adjusted results are presented in tables. The limb symmetry index (LSI) was calculated as the ratio between the injured and the uninjured Achilles tendon for the heel-rise endurance test. No sample size calculation was performed, as the impact of DVT on patient outcome is previously unknown. The level of significance was ≤ 5% for all analyses.

## Results

### Incidence of venous thromboembolic events

The specific localizations of the thromboses at 2 and 6 weeks can be found in Table 2. At 2 weeks, 76/250 patients (30%) were diagnosed with a DVT. One thrombus (1%) was noted in the popliteal vein, 43 thrombi (47%) were observed in the distal veins and 48 thrombi (52%) were recorded in the muscle veins.

**Table 2:** Localization of thromboses at 2 and 6 weeks

	Patients with positive findings at week 2 *n* (%)	Patients with positive findings at week 6 *n* (%)
Isolated proximal thrombus	0 (0)	3 (3)
Isolated distal thrombus	27 (40)	46 (40)
Isolated calf muscle thrombus	33 (45)	40 (36)
Combined proximal and distal thromboses	1 (1)	1 (1)
Combined distal and calf muscle thromboses	15 (14)	22 (20)
Total patients with thrombus	76 (100)	112 (100)

Thromboses were categorized as proximal if located in or above the popliteal vein, distal if located in the deep veins of the tibialis posterior or fibularis vein and as a muscle vein thrombosis if located in perforator veins of the lower leg. There was no screening for pulmonary embolism and could therefore only be reported if clinically presented

At 6 weeks, 112/246 patients (46%) were found to have a DVT. Four thromboses (3%) were noted in the popliteal vein, 69 (51%) in the distal veins and 62 (46%) in the muscle veins.

The cumulative DVT incidence within 6 weeks of leg immobilization was 49% (122/251).

Newly diagnosed propagation and regression of thrombi between 2 and 6 weeks are described in Table 3. There were no symptomatic pulmonary embolisms reported during the follow-up period.

**Table 3:** Thrombus formation, propagation and regression between 2 and 6 weeks

Formation, new patients with thrombus, *n*/*o*, (% increase)	46/76 (61)
Proximal thrombus	4/1 (400)
Distal thrombus	26/43 (60)
Calf muscle vein thrombus	14/48 (29)
Propagation of thrombus, *n*/*o* (%)	5/76 (7)
Regression of thrombus, *n*/*o*, (%)	6/76 (8)

Thrombus formation was defined as a patient without positive findings at 2 weeks, who had positive findings at 6 weeks. Propagation was defined as a patient with a more distal thrombus at 2 weeks, which was identified in a more proximal vein at 6 weeks. Regression was defined as a patient with a positive finding at 2 weeks, which had disappeared at 6 weeks

*n *number of patients with new thrombi at 6 weeks. *o* number of patients with thrombi at 2 weeks

### Patient-reported outcome

**Fig. 2:**
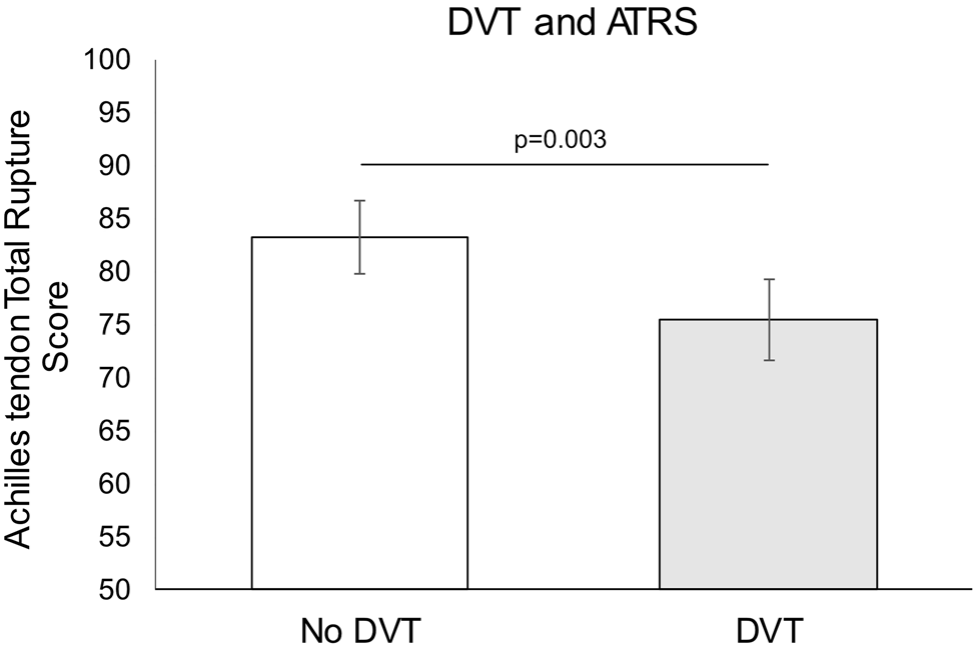
Comparison of the ATRS in the non-DVT and DVT group. Presented results are fully adjusted with 95% CI.* DVT* deep venous thrombosis

Exhibiting a DVT was associated with a significantly lower ATRS at 1-year post-surgery (mean = 76 (95% CI 72–79) vs. (mean = 83 (95% CI 80–87) (*p* < 0.01) (Fig. 2). Moreover, patients sustaining a DVT exhibited significantly more symptoms in nine of the ten subcategories of the ATRS questionnaire compared to the patients devoid of DVT (Table 4).

**Table 4:** Subcategories of the ATRS questionnaire

Variable	No DVT	DVT	Unadjusted *P*-value	Adjusted *P* value
ATRS question regarding…				
Mean (95% CI)				
…Limited in calf strength	7.6 (7.2–8.1)	6.7 (6.3–7.2)	< 0.01	< 0.01
…Tiredness in the calf	8.2 (7.8–8.6)	7.4 (7.0–7.8)	0.01	0.01
…Stiffness in the calf	7.9 (7.4–8.3)	7.1 (6.6–7.6)	0.12	< 0.05
…Pain in the calf, median (IQR)	10.0 (1)	9.8 (2)	0.06	n/a
…Every-day life activities, median (IQR)	10.0 (1)	9.0 (2)	< 0.05	n/a
…Walking on uneven surfaces, median (IQR)	10.0 (1)	9.0 (2)	< 0.01	n/a
…Walking in stairs/hills	8.8 (8.4–9.2)	8.1 (7.7–8.5)	< 0.05	< 0.05
…Running	7.7 (7.2–8.3)	6.6 (6.1–7.2)	< 0.01	< 0.01
…Jumping	7.2 (6.7–7.8)	6.0 (5.4–6.6)	< 0.01	< 0.01
…Hard physical labor	8.8 (8.4–9.2)	7.9 (7.5–8.3)	< 0.01	< 0.01

The table demonstrates differences in each of the subcategories in the Achilles tendon Total Rupture Score questionnaire. Parametric variables are presented with both and adjusted significance levels after controlling for confounding variables (patient characteristics, duration of operative time and postoperative treatment)

*ATRS *Achilles tendon total rupture score, *IQR *interquartile range

According to FAOS, patients suffering a DVT experienced worse quality of life compared to patients with no DVT (mean = 68 (95% CI 64–72) vs. mean = 75 (95% CI 71–79); *p* =  0.01). The other categories in FAOS were not significantly different between the groups.

**Fig. 3:**
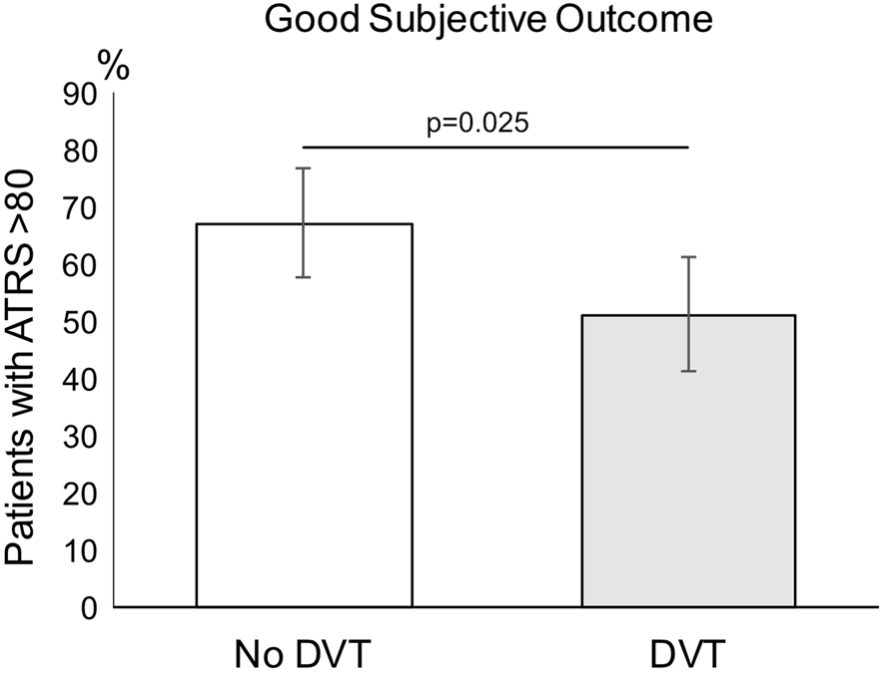
Percentage of patients with good outcome (ATRS > 80) at 1 year, compared to patients with and without a DVT postoperatively. Presented results are fully adjusted with 95% CI. *DVT *deep venous thrombosis

A significant difference in the rate of good subjective outcome (ATRS >  80) at 1 year after ATR was observed between patients with and without a DVT. Only 51% (95% CI 41–61%) (*n* =  47) of the patients suffering from a DVT exhibited a good outcome, as compared to 67% (95% CI 57–77%) (*n* =  67) of the patients not sustaining a DVT (*p* =  0.025) (Fig. 3).

### Degree of physical activity

Patients sustaining a DVT exhibited a greater loss in physical activity score (mean = 0.86 (95% CI 0.62–1.1) compared to the patients devoid of a DVT (mean =  0.56 (95% CI 0.35–0.75)), but the data were not statistically significant (*p* =  0.065).

### Calf-muscle endurance test

There was a statistically significant difference between the two groups in total concentric work, demonstrating a higher total concentric work in non-DVT patients. However, there were no statistical differences between the DVT and the non-DVT group in terms of the other functional outcome measurements (Table 5).

**Table 5:** Results from the muscle endurance test in the non-DVT and DVT groups

Variable	No DVT	DVT	Unadjusted *P* value	Adjusted *P* value
Conc. power, mean W (95% CI)	36 (34–38)	35 (33–37)	0.18	n.s
Conc. power—LSI, % (95% CI)	83 (79–87)	81 (76–85)	0.39	n.s
Tot. conc. work, median kJ (IQR)	1.9 (0.9)	1.6 (1.0)	< 0.01	n/a
Tot. conc. work—LSI, median % (IQR)	71 (28)	70 (35)	0.38	n/a
Number of rep., median n (IQR)	26 (11)	25 (12)	0.25	n/a
Heel-rise rep.—LSI, median % (IQR)	87 (25)	84 (31)	0.35	n/a
Max. height—injured leg, mean cm (95% CI)	11 (10–11)	10 (10–11)	0.02	n.s
Max. height—LSI, % (95% CI)	82 (79–86)	80 (77–84)	0.06	n.s
Ave. height—injured leg, mean cm (95% CI)	8.4 (8.0–8.9)	8 (7.7–8.6)	0.12	n.s
Ave. height—LSI, % (95% CI)	81 (78–85)	79 (75–82)	0.44	n.s

The test measured: total concentric work, total concentric power, number of heel-rise repetitions, maximum and average heel-rise height in the injured and uninjured leg. LSI represents limb symmetry index between the injured and uninjured limb. *P* values indicate significance level between groups both as unadjusted an after controlling for patient characteristics (age, BMI, sex), smoking, duration of operative time and postoperative treatment

*W *Watt, *Tot. conc. *total concentric, *LSI *limb symmetry index, *kJ* kilo joule, *IQR *interquartile range

## Discussion

The most important finding of the present study was to demonstrate that suffering from a DVT during the time of leg immobilization is associated with poorer subjective outcome in patients with an Achilles tendon rupture. Thus, patients not afflicted by a DVT during leg immobilization exhibit significantly better patient-reported outcome of the validated ATRS questionnaire, improved quality of life as assessed with the FAOS questionnaire and exhibit a higher rate of good subjective outcome.

The primary finding of this study established a significantly better long-term patient-reported outcome in patients with an Achilles tendon rupture without as compared to patients with DVT. This conclusion was substantiated by the finding that patients without DVT showed a significantly higher rate of good subjective outcome compared to the patients suffering a DVT during the postoperative leg immobilization and is also supported by a recent publication [2].

The incidence of DVT in this study was 49%, which is in good agreement with recent publications, demonstrating a 36–50% DVT rate regardless of operative or conservative treatment of the ATR [21, 25, 39]. The observation that the majority of the thromboses found were distal DVTs and/or muscle vein thromboses should be attributed to the fact that patients with high risk of venous thromboembolism were excluded from the trial and that the mean age of the included patients was relatively low, i.e., 41 years.

The high DVT incidence observed may consist of both symptomatic and asymptomatic thromboses and it is therefore suggested that even asymptomatic distal DVTs affect patient-reported outcome after ATR. The incidence of symptomatic DVTs was not registered in the trials, since it is hard for clinicians after ATR surgery to differentiate between the pain of a symptomatic lower leg DVT and the postoperative pain, which may linger after the operative procedure [9, 21].

The observed negative effects of DVT on patient-reported outcome after leg immobilization may be explained by both a stasis of the venous system and additionally by subsequently reduced arterial blood flow hindering the tendon healing potential. Microcirculation during ATR healing has been demonstrated as an essential predictor of patient outcome [28].

An additional explanation could be that low-molecular weight heparin (LMWH) administered to patients with DVT may have a negative impact on tendon healing as have been shown in animal studies [38]. This hypothesis, however, needs further investigation. Suffering a DVT might also have psychologically negative effects, as this is generally known to be a potentially lethal disease. Qualitative studies on how DVT effect patients’ psychological health are lacking, but needed to fully understand whether the results presented in this study reflect a decrease in primarily physical or psychological health.

Propagation of thrombosis is feared for and known to occur in around 10% of cases of distal DVTs [31] and in 16–25% of the patients with calf muscle thrombosis [14, 18]. The observation of only 7% propagation may be explained by the fact that all patients diagnosed with DVT or isolated calf muscle thromboses at 2 weeks received LMWH treatment in full dosage when first detected, which may result in the reduction of thrombosis progression [15].

Factors that may increase the risk of DVT, but possibly also negatively modify ATR healing, such as patient age, BMI, postoperative treatment and duration of operative time, were additionally investigated [4, 6, 17, 27, 34]. Age have been demonstrated to increase the risk of venous thromboembolism (VTE) by 2% per increase in year [8, 29]. Prolonged duration of operative time more than 60 min increases the risk of thrombosis according to NICE [1], but shorter duration of operative time after ATR has been demonstrated to hamper both subjective and functional outcome [34]. The observation that all these factors did not affect the reported results therefore corroborates the main results, showing that DVT has a negative effect on the patient-reported outcome.

The finding that half of the patients in this study sustained DVTs, which impair their subjective outcome, adds knowledge to the ongoing debate about thromboprophylaxis in lower leg immobilized patients [5]. None of the patients included in these trials initially received DVT prophylaxis, in accordance with international guidelines [1, 11, 12]. Low-molecular weight heparin has shown to be low to non-effective for DVT prevention after ATR [11, 21], presumably due to deficient blood flow and subsequent low drug concentration in the immobilized limb [7]. A suggested alternative to pharmacological prevention is mechanical DVT prophylaxis, which specifically targets the venous stasis during leg immobilization using intermittent pneumatic compression devices [9, 11, 16]. Further studies to prevent DVT during outpatient leg immobilization are warranted, possibly by using intermittent pneumatic compression.

The clinical importance of, mostly asymptomatic, DVT during lower leg immobilization has been described as an important area of research in the latest Cochrane review available [39]. The results described in this study demonstrate that both clinically asymptomatic and symptomatic DVTs impair patient outcome after lower leg immobilization.

One potential limitation of our study is the retrospective cohort study design. Controlling for potential confounding factors, however, strengthens the significance of the results demonstrated in this study. This was, however, not possible with variables demonstrating a non-parametric distribution. There was a significant difference in patient age in the baseline characteristics, which was adjusted for in all parametric analyses. However, adjusting for confounding factors cannot be done for non-parametric variables. Patient age might therefore explain the observed difference in, e.g., concentric work better than the incidence of DVT, as patient age is a known factor to decrease the calf endurance of the patients [37].

## Conclusion

Patients with an Achilles tendon rupture exhibit a high incidence of DVT, which significantly affects the subjective patient outcome. Patients suffering a DVT overall exhibit more symptoms, do not attain a good outcome and experience a lower quality of life compared to patient not suffering a DVT. Novel and more efficacious DVT prophylaxis regimens during lower limb immobilization may therefore prove important not only for the prevention of pulmonary embolism and mortality, but additionally to limit patient morbidity and to improve patient outcome.
